# The Association Between Melasma and Postinﬂammatory Hyperpigmentation in Acne Patients

**DOI:** 10.5812/ircmj.5358

**Published:** 2013-05-05

**Authors:** Hassan Adalatkhah, Homayoun Sadeghi Bazargani

**Affiliations:** 1 Department of Dermatology, Ardabil University of medical sciences, Ardabil, IR Iran; 2 Department of Statistics and Epidemiology, Tabriz University of medical sciences, Tabriz, IR Iran

**Keywords:** Acne Vulgaris, Melanosis, Hyperpigmentation

## Abstract

**Background:**

Although, melasma is most prevalent among Asian young women, and also darkly pigmented individuals are particularly prone to developing post inflammatory hyperpigmentation, to the best of our knowledge, there are rare or no studies about the association of melasma and Post inflammatory hyperpigmentation.

**Objectives:**

The aim of this study was to investigate how likely is a melasma patient to developed post inflammatory hyperpigmentation when compared to patients with inflammatory acne lesions who do not have melasma.

**Patients and Methods:**

This comparative study was conducted on 400 participants, 200 subjects involved with pigmented lesions of melasma and inflammatory acne lesions and200 involved only with inflammatory Acne lesions without melasma. Melasma, acne and post inflammatory hyper pigmentation, if existed, were assessed by a dermatologist, and pigmentation depth was assessed by wood's lamp. Multivariate logistic regression analysis suitable for study design was used to assess the association between melasma and post-acne pigmentation.

**Results:**

We found out that 24.1% of patients without melasma had post-acne pigmentation compared to 66.8% in melasma group (P < 0.001). The likelihood of observing post-acne pigmentation was found to be nearly six times more in melasma patients versus those without melasma. Association existed after controlling for possible confounders such as melanin score and time length of self-reported sun exposure, and acne severity score.

**Conclusions:**

Melasma appears to increase the likelihood of post-acne pigmentation.

## 1. Background

Knowing about the dermatologic disorders that are more specific to different ethnic groups, is not only necessary for dermatologists working in countries out of US and Europe, but it is also a must learn for clinicians working in the US and the European countries with high immigration rates. According to the changing pattern of demographics in the US population, it is estimated that various ethnic subgroups will comprise a major share of the US population (1). Acne vulgaris is stated to be among the leading causes of skin diseases in the ethnic groups of patients who seek for treatment (2). Moreover, the pigmentary disorders are among the disorders with high variety across populations and such disorders can be psychologically distressing because of their visible nature (1). Melasma is known to be a prevalent acquired pigmentary disorder of the face and is a symmetrical hypermelanosis with slow progression: irregular coloration and irregular outline. It is most prevalent among young to middle aged women with darker skin phototypes (Asian or African or Middle Estern). Studies assessing histology of the diseases have identified that melanin increases in epidermis, dermis, or both of them. Melanocytes show increased activity leading to increased formation of melanosomes, their melanization, and transfer to the superficial epidermis and dermis layers (3). Postinﬂammatory hyperpigmentation (PIH) is a common type of acne scarring. It is a discoloration mostly left after previous acne lesion in black or brown color. These lesions are more common in patients with darker skin (4). PIH represents an acquired excess of melanin pigments following cutaneous inflammation or injury such as acne, contact dermatitis, atopic dermatitis or trauma. Darkly pigmented individuals are particularly prone to developing this form of hypermelanosis (3). Also, the hypermelanosis is identified clinically to last longer in darker-pigmented individuals than in other people (5). Several chemical mediators appear to play a role in PIH. Arachidonic Acid metabolites such as prostaglandin E2, leukotriene (LT) C4, LT D4 and thromboxane B2 are known to stimulate melanocyte by increasing the amount of immune-reactive tyrosine, a melanin Forming enzyme (6). Although, melasma is most prevalent among Asian young women, and also darkly pigmented individuals are particularly prone to developing post inflammatory hyperpigmentation, there are rare or no surveys about the association of melasma and post inflammatory hyperpigmentation.

## 2. Objectives

The aim of this study was to investigate how likely is a melasma patient to developed post inflammatory hyperpigmentation when compared to patients with inflammatory acne lesions who do not havemelasma.

## 3. Patients and Methods

This comparative study was conducted on 400 participants, 200 subjects involved with pigmented lesions of melasma and inflammatory acne lesions, 200 involved only with inflammatory Acne lesions without melasma. Study was conduct in 2008 at a dermatology clinic in Ardabil district of Iran. All subjects were women and matched for age groups. Melasma, acne and post inflammatory hyper pigmentation, if existed, were assessed by a dermatologist, and pigmentation depth was assessed by wood's lamp. Subjects were asked about history of using any drugs, excoriation and manipulation of lesions, and treatments received before the evaluations. Phototype and melanin measure were assessed using mexameter (Multi Skin Test Center, Model MC 750 and 900 English 10/2007 DK). The collected data was analyzed using state 11 statistical software packages. Multivariate logistic regression analysis suitable for study design was used to assess the association between melasma and post-acne pigmentation. Study was approved by the regional committee of ethics in Ardabil University of medical sciences.

## 4. Results

### 4.1. Background Measures

Mean age of the participants was 28.7 (SD = 5.3) years. Fifty-seven percent of the patients were married. Oral contraceptives were used by 193 (48.5%) of the patients. Seven patients had used spironolactone. No one had used phenytoin before. No previous history of Addison’s disease or Cushing’s syndrome was identified among the patients. Only one patient was found with previous history of hyperthyroidism. Cheeks were the most common place of melasma involvement with90.1% followed by forehead with 73.4%, upper lip area with 67.3% and nose with64.8% of melasma patients. Mandible and chins were involved in less than 16% of cases.

#### 4.1.1. Acne

Regarding acne status, mean GAGS score was 23.5 overall. Mean GAGS score was 22.9 for melasmatic patients versus 24 among non-melasma group (P < 0.05). Pustules were observed in 79.4% of non-melasma patients versus 68.3% in melasma patients (P < 0.05). Although percentages of patients with nodules was lower in melasma group (35.2%) than in non-melasma (40.2%) group but the difference was not statistically different. Freckle or lentigens were more common among melasmatic patients (48.2%9) than non-melasma patients observed in 30.7% (P < 0.05). Compound and intradermal nevi were observed in 40.7% of participants not significantly different between the groups. Distribution of the phototypes between groups were statistically different (P < 0.01) details of which is given in Table 1. Mean melanine score measured by mexameter was 15.9 for the non melasma group versus 19 in melasma group (P < 0.001).

**Table 1.** Distribution of the Photo Types Between Melasma and Non-Melasma Groups

Group ^a^	Phototype 2	Phototype 3	Phototype 4	Total
**Non-** **melasma** **, No. (%)**	53 (26.90)	108 (54.82)	36 (18.27)	197
**Melasma** **, No. (%)**	22 (11.1)	104 (52.3)	73 (36.7)	199
**Total**	75 (18.9)	212 (53.5)	109 (27.5)	396

^a^Pearson chi2 (2) = 25.4, P < 0.001

#### 4.1.2. Post-Acne Pigmentation

**Figure 1.**
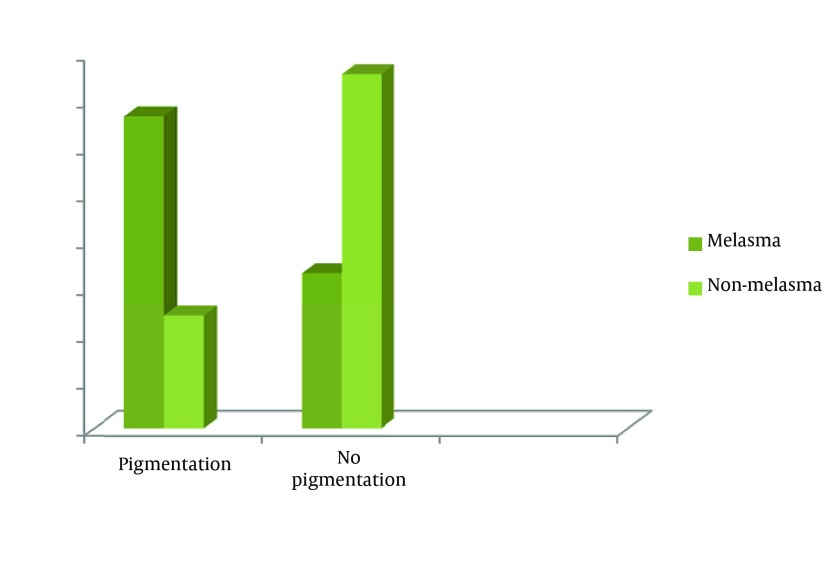
Frequency of Subjects With Post-Acne Pigmentation Compared Between Melasma Patients and Those Without Melasma

Regarding the main outcome of interest, 45.5% of all patients were found to have post-acne pigmentation. As illustrated in Figure 1, post-acne pigmentation was more prevalent among patients suffering from melasma. It was found that 24.1% of non-melasma patients had post-acne pigmentation compared to 66.8% in melasma group (P < 0.001).

The likelihood of observing post-acne pigmentation was found to be nearly six times more in melasma patients versus those without melasma. The odds ratio (OR) was calculated in bivariate analysis to be 6.3 (95% exact CI: 4-10.1). The odds ratio adjusted for possible confounding effects through logistic multivariate regression was estimated to be 5.7 (95% CI: 3.4- 9.6). Higher melanin score and average time length of self-reported sun exposure was another independent predictor increasing the odds of observing post-acne pigmentation in multivariate analysis. Nevertheless, patients who reported to have a sensitive skin to cosmetics or those having photosensitivity were found to be less likely of having post-acne pigmentation.

## 5. Discussion

The actual pathogeneses of PIH remains unknown. However, normal biologic phenomena, is more likely to have a role. This is especially noticed for release of inflammatory mediators and cytokines from both inflammatory and epidermal cells, and also the melanocytes (1). In-vitro studies have found that cytokines and inflammatory mediators like leukotriene B4, prostaglandins, endothelins and interleukins 1 and 6, and tumor necrosis factor-α, may affect the melanocytes (7). Moreover, melanogenesis may be decreased by factors like leukotriene C4. Factors like leukotriene C4 and transforming growth factor-α, may cause melanocytes to migrate (8). In the present study, the likelihood of observing post-acne pigmentation was found to be nearly six times more in melasma patients versus those without melasma. Association existed after controlling for possible confounders such as melanin score and time length of self-reported sun exposure, and acne severity score. The association of melasma with other hypermelanosis states is reported in literature. Existence of freckles, lentigines and melanocytic nevi count above three is shown to be positively related to melasma in women. The likelihood of this may be several times higher regarding melanocytic nevi (9, 10). Freckles are shown to have an autosomal transmission and its coincidence with melanocytic nevi and similar etiologic sources may explain the association between melasma and hyperpigmentation states like some types of nevi (11). The scenario may be different in case of association between melasma and post-acne pigmentation. Melasma and post inflammatory hyperpigmentation may exhibit epidermal, dermal, or mixed melanotic involvement. However, inflammation plays a major role in post inflammatory hyperpigmentation like post-acne pigmentation. PIH is caused by one of the two mechanisms that give rise to epidermal melanosis or to dermal melanosis. Arachidonic acid is released as a consequence of an inflammatory response in epidermis and is later oxidized to prostaglandins, leukotrienes or similar products to which later affect the activity of melanocytes. Particularly such products stimulate epidermal melanocyte which leads to higher levels of melanin synthesis. This in turn causes more pigments to move towards keratinocytes (12). Generally, the pathogenesis of post-acne pigmentation includes an increase in melanocyte activity and transfer of melanin granules to surrounding kerationcytes: In addition, accumulation of melanophages in the upper dermis (12). Common hormonal pathways may be suggested as an explanation for this association. For instance, due to higher number of the cytosolic and estrogen receptors in melanocytes higher sensitivity of the melasma patients to stimulation provoked by hormones is anticipated (13). The number of melanosomes is reported to increase in the keratinocytes of melasma skin densely packed more than in keratinocytes of normal skin. Therefore, it is likely that both synthesis of melanosomes in melanocytes and transfer of melanosomes to the keratinocytes are increased (14, 15). As the pathogenesis of melasma itself remains largely unknown (14), it would be difficult to define a common etiologic pathway or a causal association between melasma and post-acne pigmentation unless vast focused basic and clinical research is conducted both to confirm existence of such association and its plausible mechanisms. However, if the association between melasma and post-acne pigmentation is confirmed through the future research, it will be valuable to inform the melasma patients about their higher risk of developing post-acne pigmentation. This along with proper education will help them prevent post-acne pigmentation.
